# Editorial of the Special Issue “Molecular Medicine Applications in Infectious Diseases: Latest Innovations”

**DOI:** 10.3390/ijms242115899

**Published:** 2023-11-02

**Authors:** Sherif T. S. Hassan

**Affiliations:** Department of Applied Ecology, Faculty of Environmental Sciences, Czech University of Life Sciences Prague, Kamýcká 129, 16500 Prague, Czech Republic; sherif.hassan@seznam.cz

The integration of molecular approaches in medicine allows for a more precise understanding of the mechanisms underlying infectious diseases, paving the way for targeted therapies, personalized medicine, and the development of new diagnostic tools. It also enables the identification of specific molecular targets for drug development, contributing to the fight against drug resistance [1,2,3]. Despite these advancements, challenges endure, and ongoing innovations continue to shape this dynamic field. Here are some notable aspects:

Hurdles:
  1. Diversity of Pathogens:
    ▪ Challenge: Infectious diseases are caused by a wide array of pathogens, including viruses, bacteria, fungi, and parasites. Each requires specific diagnostic and therapeutic approaches.
    ▪ Innovation: Developing broad-spectrum molecular techniques that can identify a range of pathogens simultaneously, thereby assisting in rapid diagnosis and treatment.
  2. Drug Resistance:
    ▪ Challenge: The emergence of drug-resistant strains poses a significant threat to traditional treatments.
    ▪ Innovation: Understanding the molecular basis of drug resistance and developing targeted therapies to overcome or prevent resistance.
  3. Diagnostic Challenges:
    ▪ Challenge: Early and accurate diagnosis is crucial for effective treatment, but traditional diagnostic methods may be slow or less sensitive.
    ▪ Innovation: Generating advancements in molecular diagnostics, including PCR-based techniques and next-generation sequencing, for faster and more precise identification of infectious agents.
  4. Vaccine Development:
    ▪ Challenge: Designing vaccines that elicit a robust immune response against rapidly evolving pathogens.
    ▪ Innovation: Employing molecular techniques for vaccine development, including mRNA vaccines (with vaccines for COVID-19 serving as a successful example), which can be quickly adapted to new variants.
  5. Global Surveillance:
    ▪ Challenge: Tracking infectious disease outbreaks globally and implementing timely interventions.
    ▪ Innovation: Molecular epidemiological tools for the real-time tracking of pathogens, aiding surveillance and control measures.
New Innovations:
  1. CRISPR Technology:
    ▪ Innovation: CRISPR-based technologies for gene editing and diagnostics. CRISPR can be used to detect specific DNA or RNA sequences of pathogens, enabling precise and rapid diagnostics.
  2. Metagenomics:
    ▪ Innovation: Metagenomic approaches aid in the direct sequencing of genetic material from clinical samples, allowing for the detection of a wide range of pathogens without prior knowledge.
  3. Antibody Therapeutics:
    ▪ Innovation: Developing monoclonal antibodies and antibody cocktails as therapeutic options. This precise targeting can enhance treatment efficacy while minimizing side effects.
  4. Immunotherapies:
    ▪ Innovation: Modulating the immune response using molecular techniques, such as enhancing the activity of T cells or using cytokines to enhance the immune system against infections.
  5. Artificial Intelligence, Big Data, and Molecular Modeling Approaches:
    ▪ Innovation: Employing artificial intelligence, big data analytics, and molecular modeling techniques for predicting disease outbreaks, identifying potential drug candidates, and optimizing treatment regimens based on molecular data.
  6. Personalized Medicine:
    ▪ Innovation: Tailoring treatments based on individual genetic factors and the molecular characteristics of an infectious agent for more effective and targeted interventions.

In conclusion, while molecular medicine has significantly advanced our understanding of and ability to manage infectious diseases, ongoing innovations are essential to address the evolving challenges, ensuring timely and effective responses to emerging threats. The integration of various technologies and a multidisciplinary approach will continue to shape the future of infectious disease control. Additionally, in tandem with these efforts, it is encouraging to see the scientific community actively working towards finding solutions despite the challenges posed by drug resistance. Recently, a crucial and timely Special Issue, “Molecular Medicine Applications in Infectious Diseases: Latest Innovations”, has been published, presenting interesting studies focusing on molecular medicine applications in the field of infectious diseases and the ongoing challenges and innovative solutions faced and developed therein. The Special Issue comprises three well-designed, original studies and three up-to-date literature review articles.

Understanding the functions of specific genes in parasites can provide insights into their biology and potentially open avenues for targeted treatments. Algarabel et al. (contribution 1) identified a potential player in *Leishmania major*, the LmjPES gene, which shares homology with the human oncogene PES1. The findings point to a potential role of LmjPES overexpression in the infectivity of *Leishmania* parasites, possibly via provoking increased cell infection rates in macrophages and enhanced inflammation in an animal model, indicating its potential as a target for developing treatments or interventions against leishmaniasis. The results contribute to our understanding of the molecular mechanisms involved in this parasite’s pathogenesis and could guide future efforts in drug development and disease control strategies.

Neuraminidase is a crucial enzyme for the influenza virus, and inhibiting its activity is a common strategy employed in antiviral drugs. The complexity of developing inhibitors that can effectively target multiple sites on the neuraminidase enzyme is no small feat. Molecular modeling is a powerful tool in drug discovery and design. In the case of antiviral drug development, understanding the structure of the target enzyme, such as neuraminidase, is essential for designing molecules that can effectively suppress its activity. Selecting the sulfonamide group in the linker for effective interaction with the unique arginine triad (Arg118-Arg292-Arg371) is a clever approach developed by Evteev and colleagues (contribution 2). In their work, molecular modeling revealed the sulfonamide group’s possible advantages in this context. This approach showcases a thoughtful strategy for designing inhibitors that could be more effective and resistant to viral mutations.

Next-generation sequencing (NGS) technologies seem to be a game-changer in HIV management, especially with regard to the Sentosa^®^ SQ HIV Genotyping Assay (Vela Diagnostics). The ability to detect drug resistance more quickly and adjust therapy promptly can make a significant difference in the lives of those living with HIV. Bonifacio et al. (contribution 3) provided a detailed evaluation related to the performance of the Vela Diagnostics NGS platform, particularly the Sentosa^®^ SQ HIV Genotyping Assay that is designed for detecting drug resistance in HIV management. Their study suggests that the investigated assay shows promise in HIV resistance testing. The technology demonstrated good performance in terms of accuracy, reproducibility, and concordance with traditional sequencing methods. However, continual optimization efforts are needed to address technical challenges and achieve a more favorable balance between costs and processing speed.

Hassan et al. (contribution 4), in their up-to-date review article, explore the therapeutic potential of plant-based non-flavonoid polyphenols in combating human herpesviruses. Their review highlights the diverse bioactivities of these natural compounds and their promise in addressing drug resistance challenges. It also discusses the molecular mechanisms supporting the therapeutic impact, suggesting that plant-based non-flavonoid polyphenols could offer a multifaceted approach to tackling herpesvirus infections.

Flavonoids, with their natural origins, seem to hold a great deal of promise in the realm of controlling virus-associated cancers. Hassan and Šudomová (contribution 5) produced an up-to-date review article that emphasizes the potential of flavonoids in combating cancers associated with gamma-herpesviruses such as the Epstein–Barr virus (EBV) and Kaposi’s sarcoma-associated herpesvirus (KSHV). Their review delves into various categories of flavonoids and elucidates their mechanisms of action, shedding light on their promising roles as therapeutic agents against cancers linked to EBV and KSHV. The primary focus of their review revolves around the capacity of these flavonoids to hinder EBV and KSHV infections as well as their ability to impede the formation and growth of tumors associated with these viruses. The mechanisms through which flavonoids exert their effects are diverse, and this review explores the intricate details of how each type of flavonoid interacts with the viruses and influences tumor development. The collective insights provided in the review offer a comprehensive understanding of the potential therapeutic benefits of flavonoids in the context of these specific malignancies, offering avenues for further research and development of novel treatment strategies.

Another review article, written by You and colleagues (contribution 6), provides a comprehensive overview of the relationship between impaired expression of the nucleotide-binding oligomerization domain (NOD)-like receptor (NLR), an intracellular pathogen-recognition receptor, and the pathophysiology of otitis media (OM) in individuals with impaired innate immunity. This review suggests that NLRs are integral to the immune response in the context of OM and that their impairment can have significant implications for the development and course of this condition. Moreover, the interplay of NLR expression with bacterial infection, fluid characteristics, disease recurrence, tissue type, and surgical intervention adds another layer of complexity to our understanding of OM. This information may contribute to a better understanding of the immunological mechanisms underlying OM and potentially inform future therapeutic strategies.

Since molecular medicine has taken the fight against infectious diseases to new heights, the Guest Editor would like to express his appreciation to all the authors who made significant contributions to this Special Issue and presented unique concepts, methods, and findings that can be used as a valuable platform for future research.
